# Supplementary material for: Exploration of Machine Learning for Hyperuricemia Prediction Models Based on Basic Health Checkup Tests
Source: J Clin Med. 2019 Feb 2;8(2):172. doi: 10.3390/jcm8020172 (PMC6406925; doi:10.3390/jcm8020172)
Supplement: Supplementary file 1 [file jcm-08-00172-s001.pdf]

Supplementary Materials: Exploration of Machine Learning for Hyperuricemia Prediction Models Based on Basic Health Checkup Tests

Table 1. Performance measures for each algorithm.

Criterion	Training Set						Test Set					
	**Accuracy**	**Sensitivity**	**Specificity**	**BCR**	**Precision**	**F1score**	**Accuracy**	**Sensitivity**	**Specificity**	**BCR**	**Precision**	**F1 Score**
**Discrimination analysis classification**												
Max sensitivity criterion	0.70	0.58	0.73	0.65	0.35	0.44	0.70	0.59	0.73	0.65	0.37	0.45
Max specificity criterion	0.80	0.00	1.00	0.00	N/A	N/A	0.78	0.00	1.00	0.00	N/A	N/A
Max F1-score criterion	0.72	0.55	0.77	0.65	0.37	0.44	0.71	0.54	0.76	0.64	0.38	0.45
Max BCR criterion	0.70	0.58	0.73	0.65	0.35	0.44	0.70	0.59	0.73	0.65	0.37	0.45
**k-nearest neighbor classification**												
Max sensitivity criterion	1.00	1.00	1.00	1.00	1.00	1.00	0.72	0.34	0.82	0.53	0.34	0.34
Max specificity criterion	1.00	1.00	1.00	1.00	1.00	1.00	0.78	0.15	0.95	0.38	0.47	0.23
Max F1-score criterion	1.00	1.00	1.00	1.00	1.00	1.00	0.72	0.34	0.82	0.53	0.34	0.34
Max BCR criterion	1.00	1.00	1.00	1.00	1.00	1.00	0.72	0.34	0.82	0.53	0.34	0.34
**Naïve Bayes classification**												
Max sensitivity criterion	0.62	0.73	0.60	0.66	0.31	0.44	0.63	0.73	0.60	0.66	0.33	0.45
Max specificity criterion	0.80	0.00	1.00	0.00	N/A	N/A	0.78	0.00	1.00	0.00	N/A	N/A
Max F1-score criterion	0.69	0.61	0.71	0.66	0.34	0.44	0.69	0.61	0.71	0.66	0.36	0.45
Max BCR criterion	0.62	0.73	0.60	0.66	0.31	0.44	0.63	0.73	0.60	0.66	0.33	0.45
**Support vector machine classification**												
Max sensitivity criterion	0.53	0.48	0.54	0.51	0.21	0.29	0.52	0.48	0.54	0.51	0.22	0.30
Max specificity criterion	1.00	1.00	1.00	1.00	1.00	1.00	0.78	0.00	1.00	0.00	N/A	N/A
Max F1-score criterion	0.53	0.48	0.54	0.51	0.21	0.29	0.52	0.48	0.54	0.51	0.22	0.30
Max BCR criterion	0.53	0.48	0.54	0.51	0.21	0.29	0.52	0.48	0.54	0.51	0.22	0.30

**Decision tree classification**												
Max sensitivity criterion	0.80	0.10	0.97	0.31	0.52	0.17	0.78	0.08	0.97	0.28	0.49	0.14
Max specificity criterion	0.80	0.00	1.00	0.00	N/A	N/A	0.78	0.00	1.00	0.00	N/A	N/A
Max F1-score criterion	0.80	0.10	0.97	0.31	0.52	0.17	0.78	0.08	0.97	0.28	0.49	0.14
Max BCR criterion	0.80	0.10	0.97	0.31	0.52	0.17	0.78	0.08	0.97	0.28	0.49	0.14
**Random forest classification**												
Max sensitivity criterion	0.78	0.88	0.75	0.81	0.47	0.61	0.68	0.66	0.69	0.67	0.36	0.47
Max specificity criterion	0.81	0.08	0.99	0.29	0.74	0.15	0.79	0.07	0.98	0.27	0.66	0.13
Max F1-score criterion	0.73	0.72	0.73	0.73	0.40	0.52	0.70	0.64	0.71	0.68	0.37	0.47
Max BCR criterion	0.73	0.71	0.73	0.72	0.40	0.51	0.70	0.64	0.71	0.68	0.37	0.47

BCR: balanced classification rate
